# Inflammaging and Brain: Curcumin and Its Beneficial Potential as Regulator of Microglia Activation

**DOI:** 10.3390/molecules27020341

**Published:** 2022-01-06

**Authors:** Antonia Cianciulli, Rosa Calvello, Melania Ruggiero, Maria Antonietta Panaro

**Affiliations:** Department of Biosciences, Biotechnologies and Biopharmaceutics, University of Bari, I-70125 Bari, Italy; antonia.cianciulli@uniba.it (A.C.); rosa.calvello@uniba.it (R.C.); melania.ruggiero@uniba.it (M.R.)

**Keywords:** inflammaging, brain, microglia, curcumin, neuroinflammation, neurodegeneration

## Abstract

Inflammaging is a term used to describe the tight relationship between low-grade chronic inflammation and aging that occurs during physiological aging in the absence of evident infection. This condition has been linked to a broad spectrum of age-related disorders in various organs including the brain. Inflammaging represents a highly significant risk factor for the development and progression of age-related conditions, including neurodegenerative diseases which are characterized by the progressive dysfunction and degeneration of neurons in the brain and peripheral nervous system. Curcumin is a widely studied polyphenol isolated from *Curcuma longa* with a variety of pharmacologic properties. It is well-known for its healing properties and has been extensively used in Asian medicine to treat a variety of illness conditions. The number of studies that suggest beneficial effects of curcumin on brain pathologies and age-related diseases is increasing. Curcumin is able to inhibit the formation of reactive-oxygen species and other pro-inflammatory mediators that are believed to play a pivotal role in many age-related diseases. Curcumin has been recently proposed as a potential useful remedy against neurodegenerative disorders and brain ageing. In light of this, our current review aims to discuss the potential positive effects of Curcumin on the possibility to control inflammaging emphasizing the possible modulation of inflammaging processes in neurodegenerative diseases.

## 1. Introduction

Aging is a significant risk factor associated with a higher incidence of some chronic conditions, including cardiovascular diseases, metabolic diseases, and not least neurodegenerative diseases. Chronic systemic inflammation is linked both with the etiology and the progression of these pathologic conditions [1].

In an attempt to identify the molecular mechanisms linked to the aging process, recent studies have hypothesized that a few closely interlinked molecular key-processes are indeed capable of influencing all others. These mechanisms include inflammation, alteration of metabolic pathways, and adaptation to stress [2]. In this context, inflammation seems to play a leading role, given that aging is characterized by an increase in the concentration of various circulating pro-inflammatory molecules, for which a new process has been defined and termed “inflammaging” [3]. 

It has been reported that inflammaging can interfere with the respiratory, cardiovascular, gastrointestinal, musculoskeletal, endocrine, and neurological systems, resulting in functional alterations, favoring the onset of age-related diseases such as neurodegenerative diseases, whose multifactorial pathogenesis remains currently not fully understood [4,5,6,7].

In light of these observations, an intervention that extends throughout the life of the individual could prove useful to counteract the risk factors linked to the onset of pathologies linked to aging [8]. 

It is now widely reported and well accepted that dietary polyphenols, including resveratrol and flavonoids, bear a protective capacity against age-related disorders, due to their antioxidant and anti-inflammatory properties and are able to attenuate cell damage associated with age [9,10,11]. Among the polyphenols, curcumin, a natural compound derived from the rhizome of Curcuma longa of the Zingiberaceae, shows pleiotropic effects exhibiting the ability to target multiple molecules including the nuclear factor kappa, an enhancer of the light chain of activated B cells (NF-κB), the main regulator of the inflammatory and oxidative pathway [12,13,14].

This review is focused on discussing the protective effects of curcumin on changes that occur during aging in the brain by analyzing the mechanisms of action involved.

## 2. Aging, Inflammaging and Brain Diseases

Aging is a physiological phenomenon directly proportional to the life span of the population, accompanied by a progressive deterioration, albeit at different rates, of the functionality of cells and organs, increasing the risk of death [15,16,17]. This is due to the onset of age-related diseases, including cancer, diabetes, cardiovascular disorders, neurovascular disorders, and neurodegenerative diseases [18,19,20].

In an effort to identify potential biomarkers of aging, several studies have pointed to nine hypothetical hallmarks divided into primary, antagonistic, and integrative traits. The main features are represented by genomic instability which is considered one of the main driving forces of aging, telomere friction, epigenetic alterations, and loss of proteostasis [15]. The antagonistic features are compensatory or antagonistic responses to primary damage and are represented by mitochondrial dysfunction, cellular senescence, and deregulated nutrient perception [21]. These responses are initially intended to mitigate harm but can eventually become deleterious themselves. Finally, the integrative characteristics are represented by altered intercellular communication and the depletion of stem cells. Integrative hallmarks are believed to be responsible for the functional decrease associated with aging due to the cumulative damage caused by primary and antagonistic hallmarks [22].

Aging, however, is a normal part, genetically programmed, of the human biological development path and various physical and biological mechanisms are involved to which are added progressive accumulation of damage caused by negative influences from the external environment [23,24].

Among the various theories that have been formulated to explain the aging process, that of immunosenescence plays a central role. This theory attributes aging to failures of the immune system, including autoimmunity and impaired defense against cancer and pathogens. The base of this process is described by a dysregulation of innate immunity, and release of circulating factors by immune cells, including cytokines which are among the molecules most responsible for homeostasis [25,26,27].

The nervous system is among the most affected by aging due to oxidative, mitochondrial and cell death alterations, which are considered common pathophysiological mechanisms of various neurodegenerative diseases such as Alzheimer’s disease (AD), Parkinson’s disease (PD), Huntington’s disease (HD) and amyotrophic lateral sclerosis (ALS). In fact, these diseases are mainly observed in elderly individuals and the risk increases with age [28]. In humans, the aged brain exhibits the usual signs of aging and is particularly vulnerable to aberrant protein aggregation and phagolysosomal system deficits, resulting in a blurred line between aging and neurodegenerative disorders [29]. As a result, many elderly adults have pathological brain anomalies that may not always correspond to their cognitive ability. This has significant implications for the treatment of patients with clinical symptoms, as well as the design of clinical trials that specifically target protein abnormalities. Given the importance of immunological responses and inflammation in brain ageing and neurodegeneration, it will become critical to distinguish between good and maladaptive attempts to preserve or repair damage to create effective therapies [30]. 

The term inflammaging, a term that combines the terms inflammation and aging, was coined in the late 1990s following a series of research on the evolution of immune mechanisms and stress on invertebrates and mammals [31,32] and refers to the connection between the processes that lead to aging and a low-intensity type of chronic inflammation. From 2000 to today, in less than twenty years, the numerous scientific studies dedicated to the theory of inflammaging have opened a new perspective on aging, putting it in close connection with the modern vision of personalized medicine [33,34]. 

The microenvironment has been described as related to inflammation and contributing to the definition of inflammaging as a complex phenotype that impacts upon a variety of age-related diseases [35,36]. Acute inflammation represents a defensive mechanism that the body can adopt, in a transitory way, to counteract dangerous situations. Once the problem is overcome, the inflammation leaves behind no consequences in our body [37]. However, when, for various reasons, the inflammatory state persists and the inflammation becomes chronic, long-standing, and self-perpetuating it may result in harmful effects [38]. Inflammaging is considered ‘sterile’ inflammation because it occurs in the absence of an obvious pathogen or foreign body [39]. The scientific literature has, in fact, highlighted a correlation between inflammaging and many chronic inflammatory diseases such as neurodegenerative diseases including PD and AD [40]. 

Inflammaging can spread from cell to cell. This diffusion is possible owing to a class of molecules called Micro RNA (MiR), known to play a pivotal role in the regulation of gene expression. They are also involved in the regulation of inflammatory pathways. In particular, three of these molecules, called MiR 21, MiR 126 and MiR 146a, known also as inflammo-miRs, have a significant pro-inflammatory effect. The inflammo-miRs have as their specific target the NF-kB pathway: a protein complex that is fundamental in regulating the immune response to infections. This pathway is an ancient form of cellular defense that regulates inflammatory processes caused by oxidation but is also directly involved in aging processes. Interestingly, the plasma levels of these MiRs vary both with age and with the presence of inflammatory diseases. For this reason, they are being studied as possible inflammaging biomarkers. In this respect, MiR21 in particular is now considered an important biomarker of inflammaging [41,42]. 

Studies carried out in centenarians have shown that people who live longer have high levels of molecules that support chronic inflammation, but at the same time also have molecules that counteract it. In centenarians, in fact, a complex and peculiar mix of pro-inflammatory as well as anti-inflammatory characteristics was found. The balance between those two conditions is likely crucial to attain healthy aging and longevity. A greater longevity would, therefore, not lie in the absence of inflammation, but in the intrinsic ability of the individual to maintain the correct balance between inflammaging and anti-inflammaging processes [35]. 

The brain is of fundamental importance in the control of crucial processes not only for life but also for processes involved in cognition and personality. The loss of brain function caused by trauma or, more frequently, by aging, is responsible for an enormous human and economic loss [43,44,45]. 

Although a common definition of normal aging has yet to be agreed upon [46], distinctive criteria for normal and pathological aging are specific signs such as central nervous system (CNS) injury, neurodegeneration, amyloid plaques, and neuropsychiatric conditions, dementia, and impaired performance on cognitive tests [47,48,49]. Physiological changes associated with brain aging have been reported to include macroscopic changes, such as cortical thinning, ventricular enlargement, accumulation of white matter hyperintensity and post-mortem weight decrease [50], glial cell count changes, axonal loss, synaptic pruning, and mitochondrial changes, up to molecular changes, including altered gene expression and epigenetic changes [51,52,53]. Brain aging is related to cognitive impairment in terms of behavior, described as cognitive aging and particularly affects cognitive domains such as information processing speed, memory, reasoning, and executive function as well as decreased well-being and an increased incidence of low mood [54,55,56]. As neurodegenerative disorders are becoming more common in older people, it follows that the need to maintain a healthy brain as we get older is becoming increasingly recognized as a societal priority [57].

**Figure 1:**
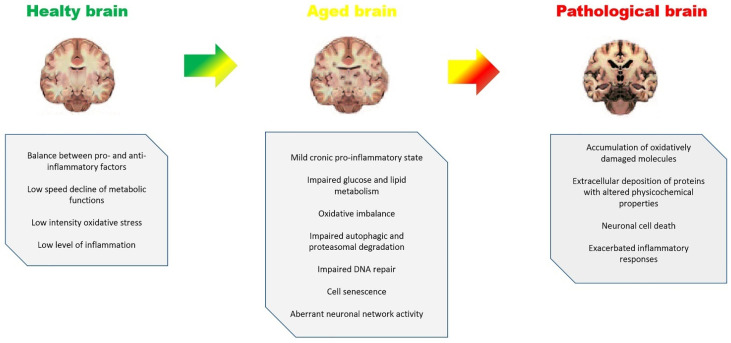
Aging brain and pathological changes which can be associated with chronic inflammation linked with neuroinflammation and neurodegeneration.

On the basis of clinical observations and behavior, it has been suggested that a number of neurological and psychiatric diseases cause premature or accelerated ageing which clearly shows a degenerative process which involves not only neurons but also glial cells such as astrocytes, oligodendrocytes, microglia, and vascular cells, interfering with their functions such as regulation of cerebral blood flow, phagocytosis, and impulse conduction [58,59,60,61]. The loss of white matter volume is roughly three times more than the loss of gray matter volume throughout the aging process. As a result, behavioral and cognitive deterioration in the elderly may be caused by changes in white matter. Furthermore, a reduction in older people’s ability to repair white matter has been discovered. In this respect it was observed that changes in white matter are seen in disorders including stroke, PD, and AD [6]. During the aging process several molecular changes occur, resulting in a complex network of connections. This makes determining whether age-related disorders are caused by a snowball effect of senescence or, conversely, the product of individual variability, an impossible task. Both explanations appear to be correct, as aging is a significant risk factor for disorders such as stroke, epilepsy, PD, AD, and brain tumors, but not in itself a specific cause [62]. In the aging brain there are many pathological changes which can be associated with chronic inflammation (Figure 1). These changes include significant decreases in dendritic and axonal arborization, dendritic spines, post-synaptic densities, presynaptic markers, synapse and cortical volume, as well as a decrease of certain neuronal populations [63].

Proliferative glial cells are a major source of neuro-inflammaging in the aging brain (astrocytes, oligodendrocytes, and microglia). Neurons depend on these cells for structural, metabolic, and trophic support [64,65,66]. However, the prolonged production of pro-inflammatory substances that occurs with increasing frequency with increasing age, which include the release of reactive oxygen species (ROS) and cytokines that attract leukocytes, can have negative consequences on nearby neurons. Recent studies suggest that senescent cells are detectable in the mammalian brain, where they could contribute to neurodegenerative processes by robust secretion of many growth factors, proteases, monocyte chemotactic proteins (MCP; aka CCL), growth-related oncogenes (GRO; aka CXCL), proinflammatory cytokines and inflammatory cytokines such as granulocyte-macrophage colony stimulating factor (GM-CSF) and inflammatory macrophage proteins (MIPs; aka CCL). Such a scenario can have powerful effects on neighboring cells and therefore can alter the local and systemic tissue environment [67,68], by altering the blood–brain barrier allowing immune cells and numerous pro-inflammatory cytokines to move into the brain parenchyma that can, in turn, modulate microglial phenotype and reactivity driving to a low-grade brain inflammation [69].

## 3. Microglia in Brain Aging and Inflammaging

Microglial cells, the myeloid cells of CNS have highly mobile, ramified pathways that allow them to patrol healthy brain tissue in a continual and dynamic manner in order to eradicate damaged neurons [70,71].

It was reported that microglial activation is a carefully controlled process. For example, increased gene transcripts in microglial cells during aging-related chronic low-grade inflammation, linked to the inflammaging process, are fundamentally different from those activated during acute inflammation, such as that caused by LPS infusion in young mice [72]. Moreover, during acute inflammation, the upregulation of genes determines a high increase in the expression of NF-κB signaling factors, whereas during inflammaging the signaling pathways related to phagosome, lysosome, or antigen presentation seems to be preferentially upregulated concurring to induce the phenotype of senescent microglia. In addition, in inflammaging, microglia show other typical morphological features as they express proinflammatory markers including IL-1β, TNFα, IL-6, and MHC-II (major histocompatibility complex class II), and contain lipofuscin granules [73]. 

Other than differences in protein expression profiles, it was also reported that in the CA1 rat hippocampus, during inflammaging, both total and reactive microglial cells decreased, and the motility of the retina decreased in aged mice [74,75]. Furthermore, while it was seen that microglial cells of young animals, after administration of ATP rapidly stretch their ramifications and increase their motility, it seems that microglial cells are much less mobile during aging [76]. Activation of microglia, which was previously thought to be a negative process that resulted in the accumulation of neurotoxic phagocytes, is now thought to be a reversible, multi-staged process that results in the generation of a variety of reactive microglial cells with protective abilities, at least in rodents [77]. 

As a result, aging may decrease and diminish the neuroprotective action of microglia. In rodents, in fact, decreased microglial migration may reduce their phagocytic effectiveness, allowing degenerating neurons and proinflammatory neuronal toxic debris to accumulate which is a typical characteristic of CNS aging [74,78]. Nonetheless, as with chronic inflammatory illnesses, amplified, exacerbated, or persistent microglial activation can result in significant pathogenic alterations and neurobehavioral consequences [79,80]. 

Inflammatory cytokines such as IL-6 and IL-1, both activators of NF-kB, recruit microglia while Interferon-γ and IL-4, released by T cells, stimulate the expression of MHC II on microglia, accelerating its proliferation [81,82]. Matrix metalloproteinases (MMPs), released from apoptotic cells, stimulate microglial activation whereas, on the contrary, IL-10 and TGF-β1 appears to negatively modulate microglia as to reduce the expression of MHC II. Activated microglia rapidly express high levels of MHC II and several other types of immunoglobulin family receptors, complement receptors, cytokines, and chemokines such as IFN-γ, IFN-β, IFN-α, IL-1, IL-6, IL-10, IL-12 and receptors for mannose. Therefore, the cells acquire the flexibility to acknowledge and bind various antigens and present them to T lymphocytes [83]. The cytoskeleton remodeling in microglial cells can be revealed using immunostaining with antibodies for IBA1, which is a microglial marker of Ca^2+^-dependent actin polymerization. In this respect it was observed that IBA1 immunostaining in microglia cells, during aging, seemed to significantly diminish in LPS rats. Conversely, microglial cells of aged rats appear to be characterized by highly short branchings, which on the contrary are extremely ramified in LPS rats [84]. 

Activated microglia then modify their form and function, producing pro-inflammatory cytokines such as IL-1, IL-6, and TNF-α switching from a protective M2-phenotype to a pro-inflammatory M1-phenotype, causing more neuroinflammation. Overproduction of pro-inflammatory mediators destroys a delicate balance, degrades synaptic plasticity, and decreases BDNF and IGF-1 production, all of which are deleterious to neural precursor cells and normal neuronal function [85,86]. 

Moreover, it was reported, in a rat model of acute inflammation, that microglia survey the brain parenchyma, probably to stop the spread of proinflammatory, damaging molecules from apoptotic neurons and debris [74,87], and reactivity that is also geared toward restoring normal physiological conditions [75,84]. 

In this regard, low-grade and chronic age-related inflammaging processes may cause or be the explanation for the defective patrolling of parenchyma microglia resulting in inefficient removal of damaged neurons compared to acute inflammatory conditions [82].

Furthermore, it has been reported that differential gene expressions of microglia in white and gray matter within the CNS was linked to different environmental conditions [88]. For example, NF-kB pathway inhibitor genes were more prevalent in white matter microglia than in gray matter microglia. Furthermore, the morphology of microglia reflects their functional capacity: different branched processes of these cells act as environmental sensors [89], regulating neurogenesis, synaptic density and connection, plasticity in normal neural tissue, as well as avoiding cytotoxicity of neurotransmitters [90].

The definition of microglia, classified as M1-neurotoxic or M2-neuroprotective on the basis of different activation patterns and cytokine production phenotypes, highlights two complementary concepts [91,92]. It is known that the transition of microglia activity from neuroprotective to neurodegenerative is dependent on time. The microglia, in fact, return to their state of “rest” every time the physiological balance of the CNS is restored [93]. Conversely, when the activity of these cells is prolonged, an overproduction of proinflammatory cytokines can exacerbate neuroinflammation by promoting neurodegenerative effects during chronic inflammation leading to neuronal damage and cognitive deficits [94]. A drastic loss of branching is observed in the aging brain, a morphological feature consistent with immunosuppression induced by persistent low-grade inflammation that develops with age identified as inflammaging. A feed-forward association between immunosuppression and inflammaging has been proposed as a possible factor in age-related diseases [95]. Microglia in aged nerve tissue, for example, display high amounts of phagocytosis markers such as CD68. However, despite their ability to execute phagocytic activity, these cells in AD showed ineffective clearance of amyloid plaques. In this respect, it is reasonable to believe that a better understanding of the processes behind altered microglial distribution and/or migration in the aged brain will lead to a major expansion of present amyloid disease knowledge [96,97,98]. 

**Figure 2:**
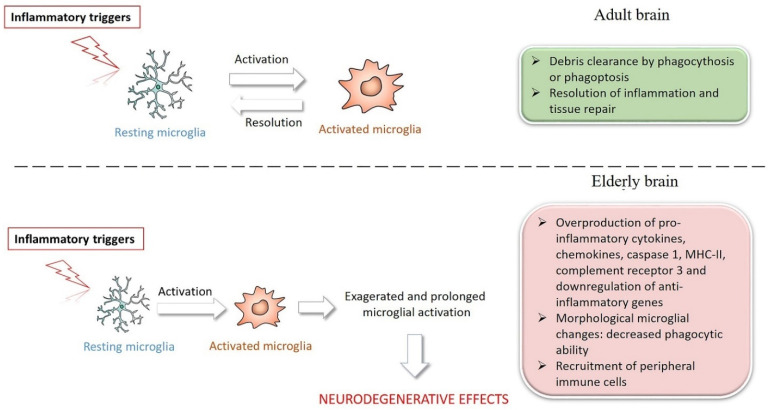
Transition of microglia activity from neuroprotective to neurodegenerative in brain aging.

Recent research reveals that microglia, such as peripheral immune cells, go through a senescence process (Figure 2). In the elderly and pathological brain, senescent and hyperactive microglia have been discovered. The aging brain, in turn, appears to be able to influence the immune system and promote immune cell recruitment from the periphery, contributing to immunosenescence and neuroinflammation. Furthermore, the age-related decrease in anti-inflammatory molecules adds to increased sensitivity to both extrinsic and intrinsic stresses [99,100,101,102]. Even in neurologically intact elderly people, overproduction of pro-inflammatory mediators in the periphery may cause a progressive increase in neuroinflammation, characterized by increased glial activation, elevated steady-state levels of inflammatory cytokines, and decreased production of anti-inflammatory molecules [85]. 

Therefore, the drug targeting of microglia appears to be a promising strategy in the regulation of neuroinflammation, redox imbalance, and oxidative stress which represent a common denominator between neurodegenerative diseases and the neuroinflammation observed during inflammaging.

## 4. Curcumin and Microglia

Microglial cells are, as previously mentioned, the resident macrophages of the CNS, that support the normal function of neurons and monitor the health of neurons in homeostasis, the resting state. Therefore, microglia display protective effects in normal conditions. In neurodegenerative diseases, such as AD, PD, ALS, or in brain injury or infection, activation of microglia plays a pivotal role by inducing oxidative stress and neuroinflammation. In fact, in these brain diseases and disorders, microglia transform into the M1 functional phenotype that release various kinds of cytokines, chemokines, reactive oxygen species (ROS), and reactive nitrogen species (RNS), which are implied in the development and maintenance of inflammatory responses. Excessive production of these inflammatory mediators could cause neuronal damage and death. Accumulated evidence suggests that suppressing microglial activation and neuroinflammation could attenuate the severity of neurodegenerative disorders [103,104,105]. 

Several natural products including curcumin, have been studied for their possible therapeutic modulatory role of microglial functions and consequent improvement of brain disorders [103]. 

Curcumin is a natural compound with a polyphenolic structure. This turmeric extract derives from the rhizome of the *Curcuma longa*, a member of Zingiberaceae, and shows a wide range of biological and pharmacological activities including antioxidant, anti-inflammatory, antimicrobial, immunomodulatory, and anti-tumor activity [106,107].

The chemical properties of curcumin have been previously well described. This compound shows three chemical components in its structure, including one diketone moiety and two more stable phenolic groups [108,109]. The stability and the effectiveness of curcumin for therapeutic applications may be influenced by several parameters of the biological environment including light exposure, temperature, and pH [108]. The active functional groups of curcumin can be oxidized via electron transfer and hydrogen abstraction processes [110]. The antioxidant activity of curcumin is determined by methylenic hydrogen and o-methoxy phenolic groups. In addition, the β-diketone groups can chelate ions with transition metals and some of these metal complexes show antioxidant enzyme-mimetic activities [111]. As previously reported, several studies were conducted to understand absorption, distribution, metabolism, and excretion of curcumin evidencing that curcumin has poor absorption and undergoes rapid biotransformation after oral administration resulting in a reduction of systemic bioavailability, determining that only traces of the compound are able to enter the systemic circulation [112,113]. A seemingly major part of this compound is that it is excreted in the feces. Curcumin has been found, indeed, to have limited penetration in the brain and testes [114,115]. 

**Figure 3:**
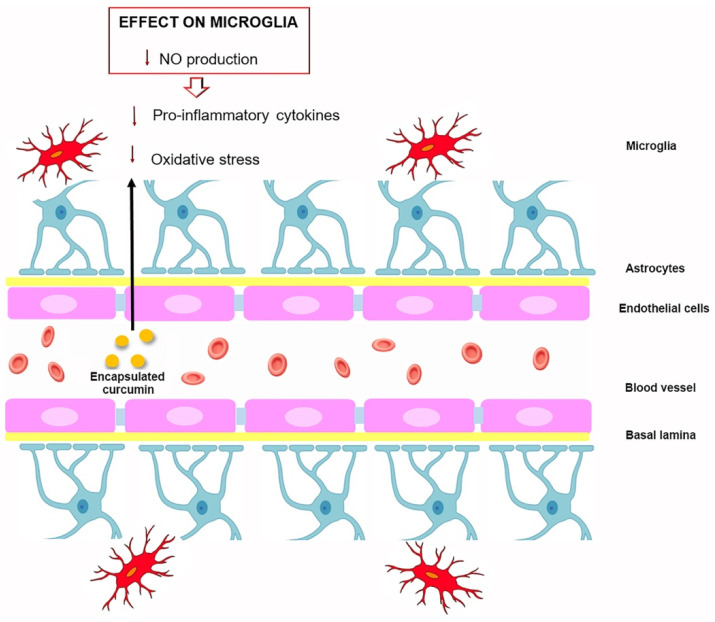
Encapsulated curcumin effects on microglia after crossing the blood–brain barrier.

The blood–brain barrier (BBB) between the blood and the CNS is able to exert the physiological function of regulating the exchange of molecules, cell infiltration and ionic homeostasis, thus preserving the brain microenvironment and protecting the CNS from circulating toxins or pathogens [116]. It is well known that the BBB is constituted of endothelial cells, which act as a protective shield by restricting entry of substances into the brain and molecules with high lipid solubility and low molecular weight easily cross this restrictive barrier into the brain. Curcumin’s poor penetration through the BBB, combined with its chemical-physical properties, pose significant limitations for the use of these new systems in brain pathologies [117]. A number of studies have been carried out to determine both the formulation of curcumin to use in the brain as well as its distribution in the brain. In this context, a study compared the distribution of conventional curcumin with that of curcumin encapsulated in polylactic-co-glycolic acid (PLGA) nanoparticles in the brain, reporting that curcumin nanoformulations concentrated more in the cerebral cortex and hippocampus and had a longer retention time [118]. Moreover, curcumin nanoformulation analysis reveals that curcumin is distributed in the liver, heart, spleen, lung, kidney and especially in the brain (Figure 3) where nanoformulations seem able to highly increase curcumin bioavailability and its mean residence time as well as its anti-inflammatory properties [119]. 

These findings provide insight into the efficacy and limitations of curcumin use as a therapeutic agent for a variety of diseases. At the same time, it also suggests that curcumin in encapsulated form, increasing its bioavailability and efficacy, can be considered an excellent tool for the treatment of various diseases, including those that are inflammatory based which trigger and worsen with the aging process.

**Figure 4:**
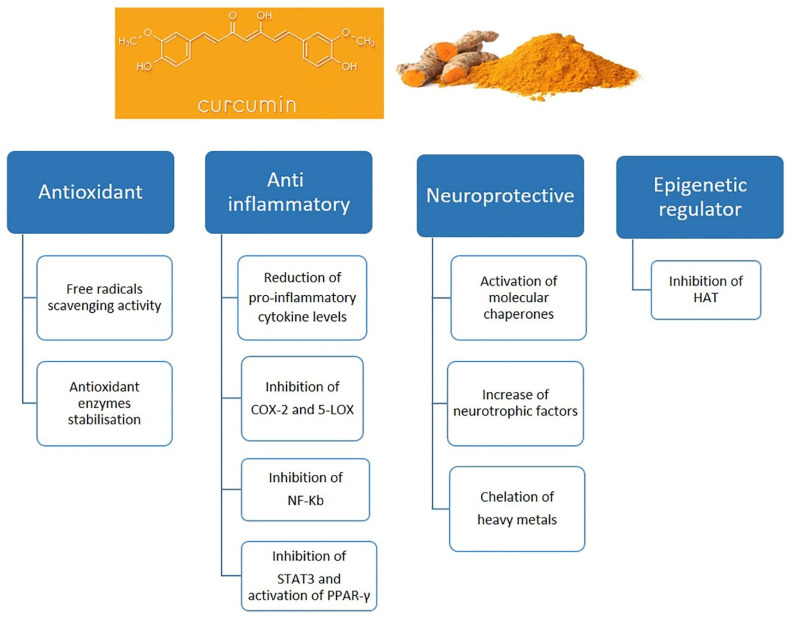
Chemical structure of curcumin and protective effects of curcumin. Abbreviations: Cyclooxygenase 2 (COX-2); 5-lipoxygenase (5-LOX); Nuclear factor-κB (NF-κB); Signal transducer and activator of transcription 3 (STAT3); Peroxisome proliferator- activated receptor gamma (PPAR-γ); Histone acetyltransferases (HAT).

In recent years, many studies have been conducted, in vitro and in vivo, which have strongly evidenced that curcumin seems to have particularly protective effects in the context of neuroinflammation (Figure 4). In the last decade, in fact, accumulating evidence suggests curcumin is a potential therapeutic agent for many diseases and disorders such viral infections, atherosclerosis, intracerebral hemorrhage, and especially neurodegenerative diseases induced by microglia [120,121,122,123,124,125]. In the brain, in fact, curcumin seems to be a pleiotropic molecule showing a possible therapeutic role on microglia inhibiting microglial transformation, modulating inflammatory mediators and thus counteracting neuroinflammation which represent the initial and critical step of neurodegenerative diseases [126]. 

### 4.1. Neuroprotective Effect of Curcumin In Vitro Studies

Curcumin, in microglial cells, interacts with multiple molecular targets including NF-κB which is a well know molecule able to exert a fundamental role in regulating the inflammatory pathway. Inhibition of NF-κB may result in the reduction of pro-inflammatory markers and consequently of the inflammation process. In this context, a study showed that curcumin blocked the LPS-mediated induction of cyclooxygenase-2 (COX2) via inhibition of NF-κB and activator protein 1 (AP1) in BV2 microglial cells [127]. 

Curcumin exerts its neuroprotective effects, other than NF-κB, also through several signaling pathways including the TLR-4 dependent signaling pathway associated with the inflammatory response. It was reported, in this respect, that curcumin could inhibit TLR-4 activation and its downstream pathway [14].

Zhu et al. demonstrated, in a co-culture system of primary neurons and microglia, that curcumin treatment after LPS stimulation significantly reduced the release of inflammatory mediators such as IL-1β, IL-6, RANTES, and suppressed microglial TLR4/MyD88/NF-κB signaling pathway protein expressions as well as neuronal apoptosis [128]. In another study, it was shown that curcumin suppresses both NF-κB-mediated pro-inflammatory stimulation and LPS-induced interferon regulatory factor 3 (IRF3) activation via MyD88 and TRIF-dependent pathways [129]. Curcumin, furthermore, has also been reported to promote the development of the M2 microglial phenotype in a HO-1 dependent manner so as to reduce iNOS induction and therefore to protect microglial cells against oxidative stress [130]. In this context, Yu et al. demonstrated that curcumin suppressed the mRNA expression of COX-2 and iNOS in BV2 microglial cells stimulated by lipoteichoic acid (LTA) in a concentration dependent manner. Moreover, the same authors showed that curcumin reduces oxidative stress and neuroinflammation in LTA-stimulated BV2 microglia through activation of HO-1/Nrf2/ARE cytoprotective mechanisms. Therefore, these effects determined by curcumin leads, definitely, to anti-neuroinflammatory effects on microglia [105]. Moreover, our work group has found that curcumin is able to increase the production of anti-inflammatory cytokines, such as IL-4 and IL-10, in murine BV-2 microglial cells treated with LPS as well as promote anti-inflammatory responses in microglia through JAK/STAT/SOCS signaling pathway modulation [131]. Our work group has also shown that curcumin is able to attenuate LPS-induced inflammatory responses and downregulate the PI3K/Akt pathway in LPS-stimulated BV2 microglial cells [12]. In addition, in another study with LPS-activated BV-2 microglia, it was demonstrated that curcumin has neuroprotective and anti-inflammatory properties also via inhibition of TLR-4, MyD88, heat shock factor-1 (HSF-1), heat shock protein 60 (HSP60), and NF-κB [132]. Moreover, the anti-inflammatory role of curcumin was demonstrated in Pam3CSK4-stimulated BV-2 microglial cells. It was, in fact, reported that curcumin (10 and 20 μM) was capable in reducing the secretion of NO, PGE and TNF-α and in suppressing mRNA expression levels of iNOS and COX-2 as well as nuclear factor erythroid 2-related factor 2 (Nrf-2), and antioxidant response element (ARE) mechanisms, via inducing activation of heme-oxygenase-1 (HO-1) [133]. In addition, neuroprotective effects of curcumin were demonstrated both in neuronal cells and in microglial cells via inhibition of apoptosis, iNOS, COX-2 and HSP60/HSF-1 expression [134]. 

In the literature there is much more evidence of the promising pharmacological properties of curcumin that are expressed as anti-inflammatory, immune-modulatory and neuroprotective effects. In this context it is well known that the main anti-neurodegenerative effect of curcumin occurs via inhibition of apoptosis, TNF-α, iNOS, RNS and COX-2 as well as activation of antioxidant genes [135]. Curcumin activates, in fact, NRF-2 and HO-1 in microglia and consequently reduces oxidative stress and neuroinflammation, and thus could be considered, for this reason, as a potential neuroprotective agent working through the NRF-2 pathway [136]. Other researchers have found that curcumin suppressed cell apoptosis, decreased the release of pro-inflammatory cytokines as TNF-α, IL-1β, and IL-6, and increased IL-10 release in LPS-treated BV2 microglial cells. Furthermore, these authors discovered that protective effects of curcumin occur via modulating miR-362-3p/TLR-4 axis through the NF-κB pathway [137]. In addition, it was seen that in some circumstances, LPS can cross the BBB mediating the release of TNF-α in microglial and neuronal cells thus inducing inflammatory responses and pro-apoptotic activity via the NF-κB and MAPK pathways. In this respect curcumin seems able to inhibit TNF-α and other pro-inflammatory cytokines determining neuroprotective effects [138]. It was also demonstrated that curcumin is able to ameliorate the anti-inflammatory and phagocytic effects in microglia cells, showing a direct regulatory effect on phagocytosis of Aβ42-peptide, as well as attenuating the inflammatory picture on PGE2-stimulated N9 cells [120].

Curcumin demonstrated anti-inflammatory effects also by determining diverse alterations in the transcriptome such as inhibition of NOS2, IL-6, and COX-2, which are all related to the NF-κB, AP-1, and STAT3 target pathways. In addition, curcumin was demonstrated to be able to reduce TLR-2 expression in resting microglia as well as to induce IL-4 and PPARα expression after microglial activation. These observations support the pleiotropic, anti-neuroinflammatory, neuroprotective, and antioxidant effects of curcumin [139]. It was additionally reported that curcumin has antioxidant and neuroprotective effects via inhibition of MyD88/p38 MAPK, while Shi and collaborators, using primary BALB/c microglia cultures, demonstrated that curcumin suppresses ERK1/2 and p38 MAPK, attenuating inflammatory responses [140,141]. In Table 1 a summary on the in vitro effects of curcumin on microglia are reported. 

### 4.2. Neuroprotective Effect of Curcumin In Vivo Studies

The beneficial effect of curcumin has also been studied in in vivo models. By using neonatal Sprague-Dawley rats, it was shown that curcumin, administrated at a dose of 100 mg/kg, intraperitoneally, was able to ameliorate white matter injury and pre-oligodendrocyte death, as well as inhibit iNOS and NOX (p67phox and gp91phox) expression in microglia [142]. Indeed, employing exosome-encapsulated curcumin in C57BL/6j mice brain inflammation in experimental autoimmune encephalomyelitis was attenuated and microglia apoptosis was induced. In this context, the intranasal administration of exosome-encapsulated curcumin was shown to be able to reduce LPS-induced brain inflammation, as well as experimental autoimmune encephalitis, and delay the growth of GL26 brain tumors in C57BL/6j mice [143]. Furthermore, the neuroprotective effects of nano-curcumin were observed in early brain injury (EBI) after experimental subarachnoid hemorrhage (SAH). Using the endovascular perforation rat SAH model the poly(lactide-co-glycolide) (PLGA)-encapsulated curcumin nanoparticles (Cur-NPs) attenuated BBB dysfunction by preventing the disruption of tight junction proteins and inhibited the inflammatory response, microglial activation, oxidative stress, and cell apoptosis [125]. 

Moreover, the protective effect of curcumin on nigrostriatal dopaminergic (DA) neurons and glial responses was reported in mice with 6-hydroxydopamine (6- OHDA)-induced Parkinson’s disease, where curcumin protected the DA neurons and reduced lesions and activation of striatal astrocytes and microglia [144]. In addition, as previously noted, HIV-1 gp-120 promoted apoptosis in cortical neurons of 1-day-old Sprague-Dawley rats which was attenuated by curcumin treatment. In this study, in fact, curcumin was able to inhibit ROS, TNF-α, and MCP-1 production in gp-120-induced microglia, thus, protecting cortical neurons [145]. Furthermore, Gao et al. found that during subarachnoid hemorrhage, curcumin could reduce neuroinflammatory response, via shifting microglia phenotype toward M2 by inhibition of the TLR-4/MyD88/NF-κB signaling pathway [146]. 

Curcumin has been shown to directly affect microglial activation and to have a strong regulatory effect on microglial responses. It was reported that curcumin, in a mouse model of ischemic stroke was able to promote microglia M2 polarization suppressing inflammation, reducing neuronal damage and consequently improving function tests [104]. Thus, it is evident that curcumin has the therapeutic potential to reduce the progression of neurodegeneration in diseases including PD and AD, by inhibiting microglia-mediated pro-inflammatory responses. 

Curcumin was also shown to delay retinal degeneration via suppression of microglial activation in retinas of rd1 mice with retinal degeneration [147]. Moreover, recently it was suggested that solid lipid curcumin particles have better neuroprotective, anti-inflammatory, and anti-amyloidogenic effects than curcumin in a mouse model of AD [148]. 

Fractalkine (FKN) promotes neuroinflammation in diet-induced models of obesity. In fact, hippocampal microglia activation with neuroinflammation and reduced neurogenesis can be induced in mice feed through fructose feeding via activation of TLR-4 and NF-κB. In these mice the FKN level and CX3CR1 expression increased, leading to neuroinflammatory conditions. In this respect, it was reported that curcumin protects the fructose-induced mice via inhibition of microglial activation and suppression of FKN/CX3CR1 up-regulation in the CNS [149]. Furthermore, Zhu and collaborators evaluated the effect of curcumin in an experimental traumatic brain injury (TBI) model in mice. These authors demonstrated that intraperitoneal administration of curcumin (100 mg/kg) post-TBI was able to reduce the number of TLR-4-positive microglia/macrophages as well as the release of inflammatory mediators including IL-1β, TNF-α, MCP-1 and RANTES, and to inhibit neuronal apoptosis. This study also showed that amelioration inflammatory damage occurred through a mechanism involving the TLR-4/MyD88/NF-κB signaling pathway in microglia/macrophages in TBI [128]. 

The anti-inflammatory and antioxidant activities of curcumin for maintaining better brain function were reported in a rat model of Gulf War Illness (GWI) where administration of curcumin ameliorated cognitive and mood function in the hippocampus by reducing occurrence of hypertrophied astrocytes, activating microglia and modulating oxidative activity [125].

In addition, attention has recently been paid to the important effect that curcumin can have on the pyroptosis process linked with microglial activation. In a recent study, in fact it was demonstrated that curcumin treatment can attenuate microglial pyroptosis improving white matter integrity as well as functional outcomes, through NF-κB signaling suppression and subsequent NLRP3 inflammasome inhibition in an in vivo model of ischemic stroke pointing out that curcumin has a direct important inhibitory effect on microglial inflammasome activation and pyroptosis [150]. Moreover, in another recent study it was reported that curcumin treatment is protective against cognitive impairments in a rat model of chronic cerebral hypoperfusion combined with diabetes mellitus by suppressing neuroinflammation, apoptosis, and pyroptosis via the regulation of the TREM2/TLR-4/NF-κB pathway in microglia [151]. 

In Table 2 a summary of the main effects of curcumin on microglia in vivo is reported.

Moreover, for more detailed information on the absorption, distribution, metabolism and elimination of curcumin as well as plasma and brain pharmacokinetics of curcumin, please refer to the recent manuscript by Hasriadi and colleagues which contains highly comprehensive information on this aspect [152]. 

## 5. Curcumin as Nutraceutical Compound in Inflammaging and Brain Inflammaging

A significant number of studies have reported the strong potentials of natural products, including the potential immunomodulatory role of curcumin. In the last decade, the beneficial role of curcumin has been better defined. Curcumin, in fact, seems to be beneficial for prevention of chronic inflammatory disorders such as cardiovascular diseases, diabetes and cancers as well as in neuroinflammatory conditions where it is able to suppress microglial activation.

It is a widely known concept that chronic inflammation is linked to chronic inflammatory disorders such as autoimmune disorders, cancer, cardiovascular, and neurological diseases. In this regard, it appears clear that an alternative approach to prevent or to treat chronic inflammatory disorders could play out by suppressing chronic inflammation [153]. 

From the studies of the last decade, it appears evident that curcumin as well as its analogs and derivatives seem to have potent and diversified pharmacological activities as well as an ability to modulate various signaling events [154]. It was demonstrated, in fact, that targeting the multiple inflammation-associated cell signaling networks and in particular PI3K/Akt, MAPKs, Wnt/β-catenin and NF-κB, with curcumin is turning out to be a new pharmacological approach useful not only in the prevention but also in the treatment of chronic inflammatory diseases typical of the inflammaging status. In this direction, it has been seen that the incidence of atherosclerosis appears to be related to diet in the aging population. Along this path, it has been shown that the long-term administration of curcumin in an old mouse model improved the health picture by ameliorating oxidative stress levels and decreasing SIRT-1 expression in the aorta of old mice fed with a high-fat diet thus indicating that curcumin might be an effective molecule useful against arteriosclerotic disease [155]. 

Moreover, curcumin appears to be a promising treatment for problems associated with reproductive aging. In this regard, it was reported that curcumin was able to ameliorate ovarian quality by modulating oxidative status, anti-aging-related sirtuin gene expression and other ovulation-related genes in an old mice model. In this study, in fact, curcumin treatment was reported to be able to increase ovarian volume and the number of follicles related to increased anti-Müllerian hormone and estrogen, as well as being able to decrease FSH serum levels. In this study, it was also reported that curcumin potentiated oocyte maturation and embryo development by decreasing oxidative stress and by elevating the SIRT-1, SIRT-3, BMP-15 and GDF-9 gene expressions [156]. 

In addition, curcumin was reported to be a promising therapeutic molecule in skin disorders associated with aging. In skin aging disorders the main role is played by the β1-integrin downregulation. In this respect, it was reported that a nanoformulation of curcumin can increase not only β1-integrin gene expression but also Bcl2/Bax ratio and NF-κB expression in fibroblast cells suggesting that nanoformulation of curcumin could be used in anti-aging and wound-healing formulations [157]. 

It was also shown that curcumin could be useful in inhibition of aging processes in skeletal muscle via up-regulation of antioxidant enzymes and by directly scavenging ROS. Curcumin treatment, in fact, was reported to be able to improve muscle mass and function of aged rats increasing plantar mass and force production in a model of aged rats [158]. 

Several studies, in addition, indicate that curcumin could prevent various age associated neurological disorders. Long-term administration of curcumin was, in fact, effective in ameliorating motor function on digits of the hand in a middle-aged rhesus monkeys’ model [159]. Curcumin supplementation was also reported to attenuate motor and cognitive function both in healthy middle-aged and in older adults [160]. Curcumin, moreover, decreased cellular inflammation-related neurodegenerative and aging processes associated with increased CDGSH iron–sulfur domain-containing protein 2 (CISD2) expression, whose expression is generally progressively decreased in the spinal cord and brain of aged mice. In this respect curcumin increased the expression of CISD2 and decreased mitochondrial impairment in LPS-challenged neural cells [161]. Curcumin seems effective also in an experimental model of AD represented by HT22 cells exposed to acrolein, where it was able to increase metalloprotease as well as A-disintegrin, and to decrease the amyloid precursor protein β-secretase [162]. Moreover, it was observed, via magnetic resonance imaging and immunohistochemical studies, that curcumin-conjugated magnetic nanoparticles can bind to amyloid plaques in mice brains [163]. In addition, the protective role that curcumin could have during inflammaging is supported by the observation that a novel curcumin formulation was able to prevent cognitive dysfunction in transgenic AD mice [164]. It was additionally reported that curcumin seems to exert its beneficial role against neuroinflammation-mediated aging in different models of AD and PD [165,166]. Moreover, it was shown that curcuminoid inhibited age-associated mitochondrial impairment in rats [167] where 100 mg/kg orally-treated curcuminoid inhibited age-associated enzymes such as cytochrome c oxidase, Complex I, NADH dehydrogenase and total ATP content. The curcuminoid, in this way, seems to suppress neuronal-NOS in mitochondria. Finally, a study carried out on mice demonstrated that curcumin, in age-related cognitive dysfunction, was able to significantly inhibit oxidative stress [168].

**Figure 5:**
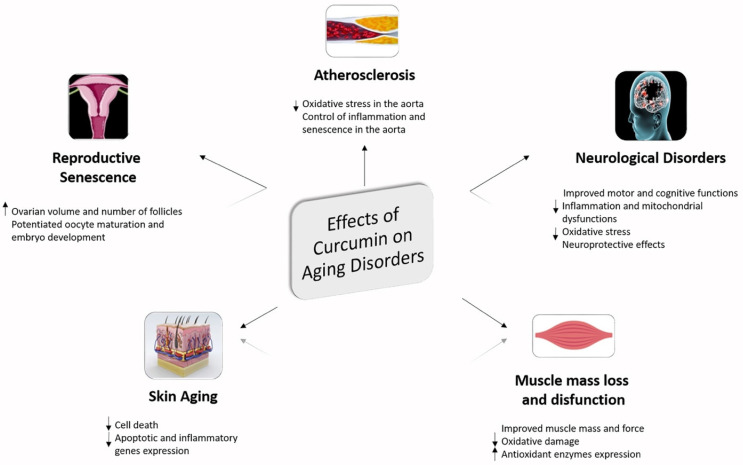
Therapeutic properties of curcumin in several aging disorders.

Curcumin’s pleiotropic activity in regulating various inflammatory mechanisms in aging models may lay the foundation for it to be proposed as a potential anti-inflammaging agent. This versatile compound, thus, seems able to suppress the molecular and cellular pathways associated with inflammation having huge potential to be used in the prevention and treatment of chronic inflammatory disorders typically observed during the aging process (Figure 5) [169]. 

Combination of standard protocols and novel therapies with natural plant extracts, including curcumin, is an auspicious approach in the prevention of development and treatment of inflammaging-linked chronic inflammatory disorders and in particular to those responsible for the progression of neurodegenerative diseases.

## 6. Perspectives 

A wide range of information and reasonable visions for prospective clinical tests as well as pharmacological therapies through aging and age-related disease in humans was reported based on data collected across investigations employing distinct aging model species.

This review focused on *Curcuma longa* as a very promising natural compound to counteract inflammaging and cognitive decline, providing deeper insight and understandings of curcumin therapeutic value in the discovery and development of new pharmacological molecules.

Curcumin as dietary phenolic compound should be considered as a pharmacological support for longevity, especially in neurodegenerative and neuroinflammatory diseases, due to its activity via declining oxidative stress, modulating signal transduction and gene expression. Curcumin, in fact, is effective as an immune enhancer in modulating systemic inflammation and brain pathologies through multiple communication mechanisms and, for this reason, is hopefully a particularly promising natural agent in counteracting the damages of aging and neurodegenerative diseases. 

In this respect, the possible interventions by curcumin on microglia suggest the possibility of this natural product to mediate regulation of microglia phenotypes and its functions and also to control redox imbalance and neuroinflammation, thus suggesting a possible use of curcumin as therapeutic agent in preventing and managing major chronic inflammatory disorders typical of the inflammaging process, including brain diseases such as AD, PD and MS.

Hence, once again, attention is paid to the modulatory potential of curcumin in its ability to mediate the anti-inflammatory effects and consequently to positively influence immunity and brain aging. Therefore, the use of curcumin as an anti-inflammatory agent with inhibitory effects on microglial transformation could be a valid and promising approach for the treatment of neurodegenerative disorders.

More clinical trials are needed now more than ever to fully establish the capacity of curcumin in the route of administration, the choice of optimal dose, and the possibilities of pharmacological responses. 

## 7. Conclusions

Multiple lines of evidence show that the involvement of microglial cells in brain neuroinflammation process typical of aging may be a target for pharmacological interventions. The anti-inflammatory activity of curcumin in microglia is ascribable to the ability of this molecule to inhibit many pro-inflammatory mediators by impeding inflammatory cascades and heightening endogenous anti-inflammatory mediators where curcumin may act as an immunomodulator. In line with what emerges from this review, there is evidence that the combination of standard protocols or new therapies with the use of curcumin-based formulations could be a desirable approach in the containment and treatment of the inflammaging process, including that related to the brain. More clinical trials are urgently required to fully establish the capacity of curcumin in the route of administration, the choice of optimal dose, and the possibilities of pharmacological responses. 

## Figures and Tables

**Table 1:** Summary on the in vitro effects of curcumin on microglia.

Cells Response	Properties of Curcumin	Reference
Reducing IL-1β, IL-6, and TNF-α expression	Anti-inflammatory	[103]
Inhibiting release of proinflammatory molecules via ERK_1_/_2_ and p38 MAPK signaling pathways	Anti-inflammatory	[141]
1. Suppression of the TLR-4-MAPK/NF-kB pathway 2. Inhibiting IRF3 activation via MyD88 and TRIF-dependent pathways	Anti-inflammatory	[129]
1. Attenuated microglia/macrophage activation and inflammatory mediators release including IL-1β, IL-6 and RANTES mediated by TLR-4/MyD88/NF-kB signaling pathway 2. Reducing c-caspase-3 expression	Anti-inflammatory Anti-apoptotic	[128]
ModulatingTLR-4 receptor and its downstream pathway	Anti-inflammatory	[14]
Ameliorates microglial phagocytosis via EP2-PKA signaling pathways	Pro-phagocytic	[120]
1. Inhibits release of NO, PGE2, and TNF-α 2. Suppresses mRNA expression of COX-2 and iNOS 3. Inhibits NF-kB and p38 MAPKs signaling and induce the expression of Nrf2 and HO-1	Anti-inflammatory Anti-oxidant	[105]
1. Increase production of IL-4 and IL-10 2. Reducing p-JAK2 and p-STAT3 expression and upregulates SOCS-1 expression	Anti-inflammatory	[131]
1. Decreased c-caspase-3 level and release of TNF-α, IL-1β, IL-6, and increased IL-10 release 2. Modulates miR-362-3p/TLR-4 axis via NF-κB pathway.	Anti-apoptotic Anti-inflammatory	[137]
1. Reducing of caspase-3, HSF-1 and iNOS expression 2. Inhibiting HSP60/TLR-4/MyD88/NF-κB signaling pathway.	Anti-apoptotic Anti-inflammatory	[132]
1. Down-regulation of the PI3K/Akt signaling 2. Reducing iNOS expression and NO production IL-β, IL-6, and TNF-α	Anti-inflammatory Anti-oxidant	[12]
Positively modulates TREM2-mediated microglial phagocytic activity	Pro-phagocytic	[136]
Inhibiting NO production via MyD88/ p38 MAPK and JNK signaling pathway	Anti-oxidant Neuroprotective	[140]

Abbreviations: IL-1β Interleukin-1β; IL-6 interleukin-6; TLR-4 Toll-like receptor 4; ERK1/2 extracellular signal-regulated Kinases ½; p38 MAPK Mammalian p38 mitogen-activated protein kinase; MyD88 Myeloid differentiation primary response protein 88; NF-kb Nuclear factor-κB; IRF3 Interferon regulatory factor 3; TRIF-TIR-domain-containing adaptor-inducing interferon-g; NO Nitric oxide; EP2 Prostaglandin receptor subtype 2; PKA Protein kinase A; TNF-α Tumor necrosis factor; HO-1 heme oxygenase-1; IL-4 Interleukin-4; IL-10 Interleukin-10; Nrf2 Nuclear factor erythroid 2; JNK2 c- Janus N-terminal kinase2; STAT3 Signal transducer and activator of transcription 3; SOCS-1 Suppressors of cytokine signaling; miR-362-3p microRNA; COX-2 cyclooxygenase 2; HSF-1 Heat shock factor1; iNOS Inducible nitric oxide synthase; HSP60 Heat shock protein 60; PI3K/Akt Phosphoinositide 3 kinase/serine/threonine kinase; TREM2 Triggering receptor expressed in myeloid/microglial cells-2.

**Table 2:** Summary of the main neuroprotective actions of curcumin on microglia in vivo.

Neuroprotective Action	Animal Model	Reference
1. Inhibiting TLR-4-positive microglia/macrophages activation and inflammatory mediators release incluging IL-1β, TNF-α, MCP-1 and RANTES and neuronal apoptosis 2. Suppression of TLR-4-MAPK/NF-kB signaling pathway	Adult male C57BL/6 mice	[128]
1. Ameliorate white matter injury and loss of preOLs 2. Inhibiting iNOS microglial expression and NOX activation	Neonatal Sprague–Dawley rats	[142]
Induces apoptosis in microglial cells of mice challenged with LPS and attenuates brain inflammation in experimental autoimmune encephalomyelitis	C57BL/6j mice	[143]
Attenuated loss of TH-fibers, diminished activation of astrocytes and microgliosis, sustained SOD1 level in the 6-OHDA-lesioned striatum	Male mice	[144]
Suppression of iNOS, TNF-α and MCP-1 in HIV-1 gp-120-induced microglia and amelioted neuronal apoptosis	Sprague-Dawley rats	[145]
1. Reduced of microglial and astrocyte activation 2. Decrease Aβ plaque formation and aberrant neuronal morphology in different brain parts	5xFAD mouse	[148]
Inhibiting microglia activation and suppressed FKN/CX3CR1 up-regulation in the brain of fructose-fed mice	Mice	[149]
Inhibited microglia activation and regulated expression levels of CCL2, ET-1, VCAM-1, TIMP-1 in the retina and improved the visual function	rd1mice	[147]
1. In CCP-treated and rescued GBM-bearing mice evokes M2 to M1 repolarization of TAM suppressing the M2-linked tumor-promoting proteins STAT3, ARG1, and IL10, and inducing the M1-linked anti-tumor proteins STAT1 and iNOS 2. Induces MCP-1 expression in TAM	Adult C57BL/6 male mice	[121]
1. Attenuates BBB disruption by preventing the disruption of tight junction proteins after SAH 2. Inhibiting mRNA levels of VCAM-1, TNF-α, MIP-2, MCP-1, ICAM-1, iNOS, IL-6, IL-1β, CINC-1, and COX-2 3. Reducing SAH-elevated MPO activity, ED-1 expression and number of ED-1 positive cells 4. Dcreasing SAH-elevated levels of ROS, MDA, 3-NT and 8-OHDG and increases SOD, GSH-Px and catalase activities 5. Suppression SAH-mediated oxidative stress	Sprague Dawley rats	[125]
1. Reduces occurrence of hypertrophied astrocytes and activated microglia, and modula oxidative in the hippocampus of GWI rats 2. Decreasing anxiety-like behavior and maintained better memory function in GWI rats.	Sprague Dawley rats	[124]

Abbreviations: MCP-1 monocyte chemoattractant protein-1; RANTES regulated upon activation, normal T cell expressed and secreted; preOLs, premyelinating oligodendrocytes; NOX superoxide-generating NADPH oxidase; LPS lipopolysaccharide; TH Tyrosine hydroxylase; SOD1 superoxide dismutase1; 6-OHDA 6-hydroxydopamine; MCP-1 monocyte chemotactic protein-1; HIV-1 human immunodeficiency virus type 1; Aβ amyloid beta protein; FKN/CX3CR1 fractalkine/C-X3-C motif chemokine receptor 1; CCL2 Chemokine (C-C motif) ligand 2; VCAM-1 Vascular cell adhesion molecule 1; TIMP-1 Tissue inhibitor of metalloproteinase 1; ET-1 Endothelin 1; CCP phytosomal curcumin; GBM Glioblastoma; TAM tumor-associated microglia/macrophages; ARG1 arginase1; BBB Blood–Brain Barrier; SAH Aneurysmal subarachnoid hemorrhage; VCAM-1 vascular cell adhesion molecule-1;MIP-2 macrophage inhibitory protein-2; ICAM-1 intracellular adhesion molecule-1; CINC-1 chemokine-induced neutrophil chemoattractant-1; MPO myeloperoxidase; ED-1 marker of activated microglia/macrophage; ROS reactive oxygen species; MDA Malondialdehyde; 3-NT 3-nitrotyrosine; 8-OHDG 8-Hydroxydeoxyguanosine; GSH-Px glutathione peroxidase; GWI Gulf War Illness.

## Data Availability

Not applicable.

## Sample Availability

Samples of the compounds are not available from the authors.
